# HNF1β, LHX1, and GGNBP2 deletion contributed to kidney and reproductive dysfunction in 17q12 deletion syndrome: evidence from a case report

**DOI:** 10.3389/fgene.2024.1391804

**Published:** 2024-08-16

**Authors:** Chun-Yu Song, Jing Yang, Sheng Jiang, Guo-Li Du

**Affiliations:** ^1^ State Key Laboratory of Pathogenesis, Prevention and Treatment of High Incidence Diseases in Central Asia, Urumqi, China; ^2^ Department of Endocrinology, First Affiliated Hospital of Xinjiang Medical University, Urumqi, Xinjiang, China; ^3^ First Clinical Medical College of Xinjiang Medical University, Urumqi, Xinjiang, China; ^4^ Bayingolin Mongolian Autonomous Prefecture People’s Hospital, Kuerle, China

**Keywords:** 17q12 deletion syndrome, MODY-5, LHX1, GGNBP2, HNF1B

## Abstract

17q12 deletion syndrome is a chromosomal abnormality, where there is a small missing piece (deletion) of genetic material on the long arm (q) of chromosome 17. Sign and symptoms can vary widely among different patients. Recently, a patient was diagnosed with 17q12 deletion syndrome in our hospital, and the clinical characteristics presented as absence of the right kidney, compensatory hypertrophy of the left kidney, multiple small cysts in the left kidney, pancreatic atrophy, hypomagnesemia, bowed uterus, multiple follicular cysts in both lobes of the thyroid gland, and maturity-onset diabetes of the young type 5 (MODY-5). A 1.5-Mb deletion with haploinsufficiency for 20 genes within the 17q12 region was found through copy number variation (CNV) analysis based on metagenomic next-generation sequencing (mNGS) technology. In addition to HNF1B absence, the LIM-class homeobox 1 transcription factor (LHX1) and GGNBP2 absence was also involved in regulation of kidney development and the reproductive system through bioinformatics analysis. The inheriting risk of 17q12 deletion syndrome is about 50%, and it is recommended to provide genetic counseling to all patients who are suspected or diagnosed with the syndrome.

## 1 Introduction

17q12 deletion syndrome is a rare chromosomal aberration, where a 1.06–2.46-Mb DNA sequence on the long arm of chromosome 17 is deleted, including AATF, ACACA, c17orf78, DDX52, DHRS11, DUSP14, GGNBP2, HNF1B, LHX1, MRM1, MYO19, PIGW, SYNRG, TADA2A, and ZNHIT3 (Edghill et al., 2024; Mitchel et al., 2024). This syndrome is associated with multiple organ systems, including kidney or urinary tract malformations, diabetes mellitus (DM), neurodevelopmental, learning disabilities, autism spectrum disorder, and schizophrenia. The exact cause and mechanisms are still to be studied (Mitchel et al., 2024). One of the major clinical features presented as maturity-onset diabetes of the young (MODY). MODY belongs to one subtype of special diabetes characterized by autosomal-dominant inheritance and early-onset insulin secretion defect (Amed and Oram, 2024). To the best of our knowledge, 14 different candidate gene mutations or deletions have been identified as MODY causes (Horikawa, 2024). As one of the mostly common subtypes of MODY, the occurrence of MODY-5 was mainly contributed to monoallelic defects in exons of the *HNF1B* gene (Horikawa, 2024; Horikawa Y Fau - Iwasaki et al., 2024). More than 50%–60% of HNF1B-related diseases involve chromosome 17q12 deletion syndrome. The clinical presentation of HNF1B-MODY varied among patients, and it is often be misdiagnosed as either type 1 (T1DM) or type 2 diabetes mellitus (T2DM). To establish an accurate diagnosis, DNA sequencing is often required. In this case report, one patient was diagnosed as 17q12 deletion syndrome in our hospital. Through DNA sequencing and bioinformatics analysis, we found that HNF1B, LHX1, and GGNBP2 absence contributed to this syndrome.

## 2 Case presentation

**FIGURE 1:**
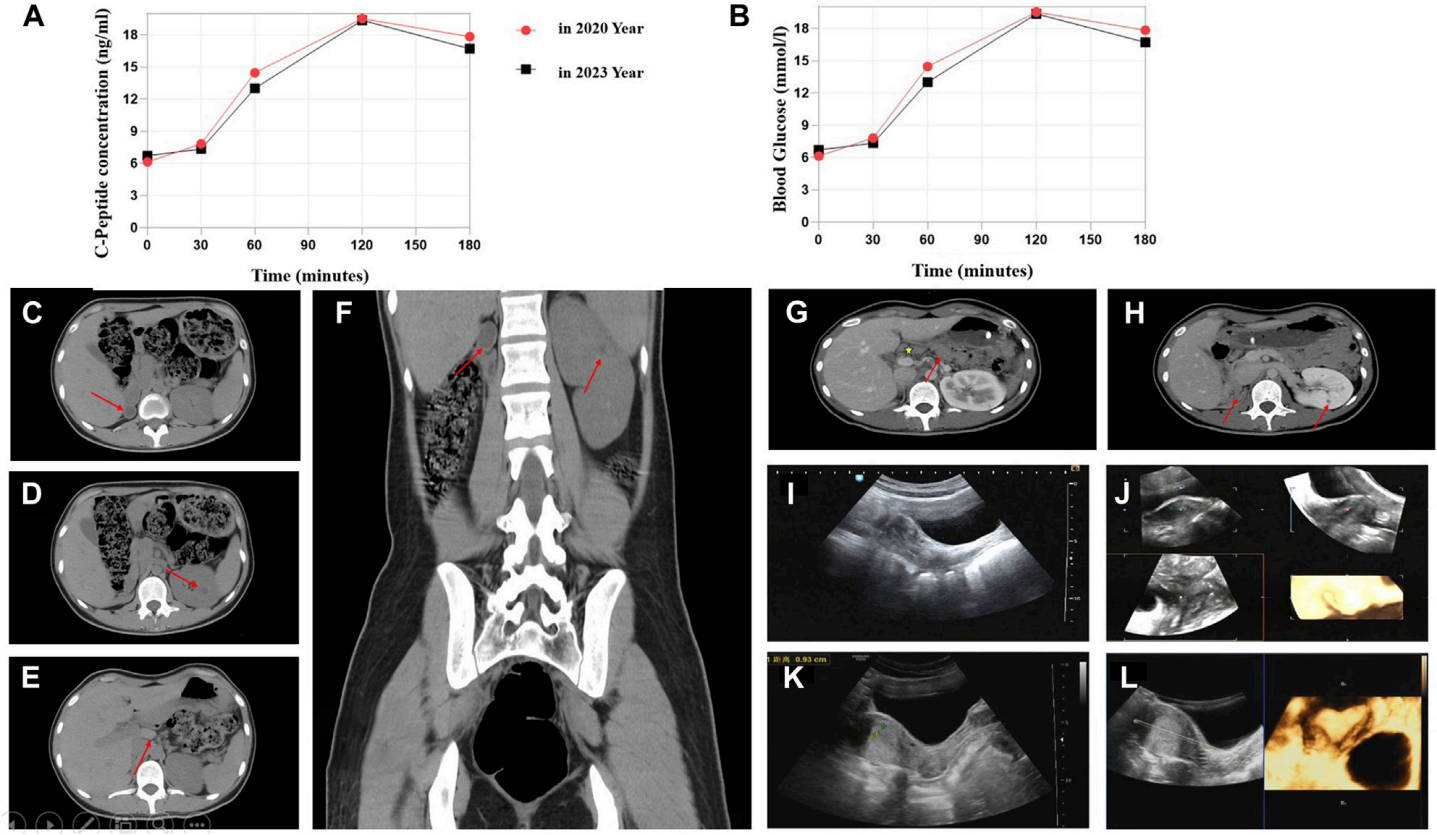
C-Peptide test, OGTT test, and imaging findings. **(A)** C-peptide concentration after glucose overload in 2020 and 2023 year. **(B)** Oral glucose tolerance test (OGTT) in 2020 and 2023. Abdominal CT image: **(C)** Right atrophied kidney (arrow). **(D)** Multiple small cysts in the left kidney (arrow). **(E)** Abnormal morphology of the pancreas with the atrophied tail part (arrow). **(F)** Enlargement of the left kidney. Contrast-enhanced CT scan: **(G)** Abnormal pancreas (arrow represents the pancreas body and atrophied tail). **(H)** Small cysts in the left kidney. Ultrasonographic view of the uterus. **(I,J)** Depression at the base of the uterine cavity with 0.6-cm uterine myometrial fundus projecting into the uterine cavity (2020 year). **(K,L)** Bowed uterus imaging result (2023 year).

The 23-year-old girl employed in this study was unmarried and childless, and admitted to the local hospital in February 2020. This patient presented with “dry mouth, polydipsia, and polyuria with weight loss for 2 months.” The concentration level of fasting venous blood glucose was 30.0 mmol/L, the serum potassium was 2.81 mmol/L, and the hemoglobin A1c (HbA1c) level was 16.8%. The urine routine test showed that urine glucose was 4 + and ketone body was 3 +. After “diabetic ketoacidosis” was cured, the patient was given four times insulin injection. In March 2020, the patient was admitted to First Affiliated Hospital of Xinjiang Medical University for the first time and diagnosed with T1DM. Four times insulin injections were continued for the treatment of DM as following: pre-prandial injections of short-acting insulin (insulin asparagus, 5IU-4IU-4IU per day) and bedtime injection of long-acting insulin (insulin glargine, 8IU per day). For self-monitoring blood glucose (SMBG), the fasting blood glucose was 5–8 µmmol/L and 2-h postprandial blood glucose was approximately 11 mmol/L; the patient had one episode of hypoglycemia, the specifics of which could not be described by the patient. In order to control blood glucose better, she visited our hospital again on 9 May 2023. The patient’s computer tomography (CT) scan showed that absence of the right kidney, compensatory hypertrophy of the left kidney, multiple small cysts in the left kidney, pancreatic atrophy (Figures 1C–H); Gynecologic ultrasound suggests that bowed uterus (Figures 1I–L). Different from the typical T1DM, the patient’s β-cell function was not very bad with low but not complete insulin secretion deficiency, and the total exogenous insulin injection dose was relatively low.

**FIGURE 2:**
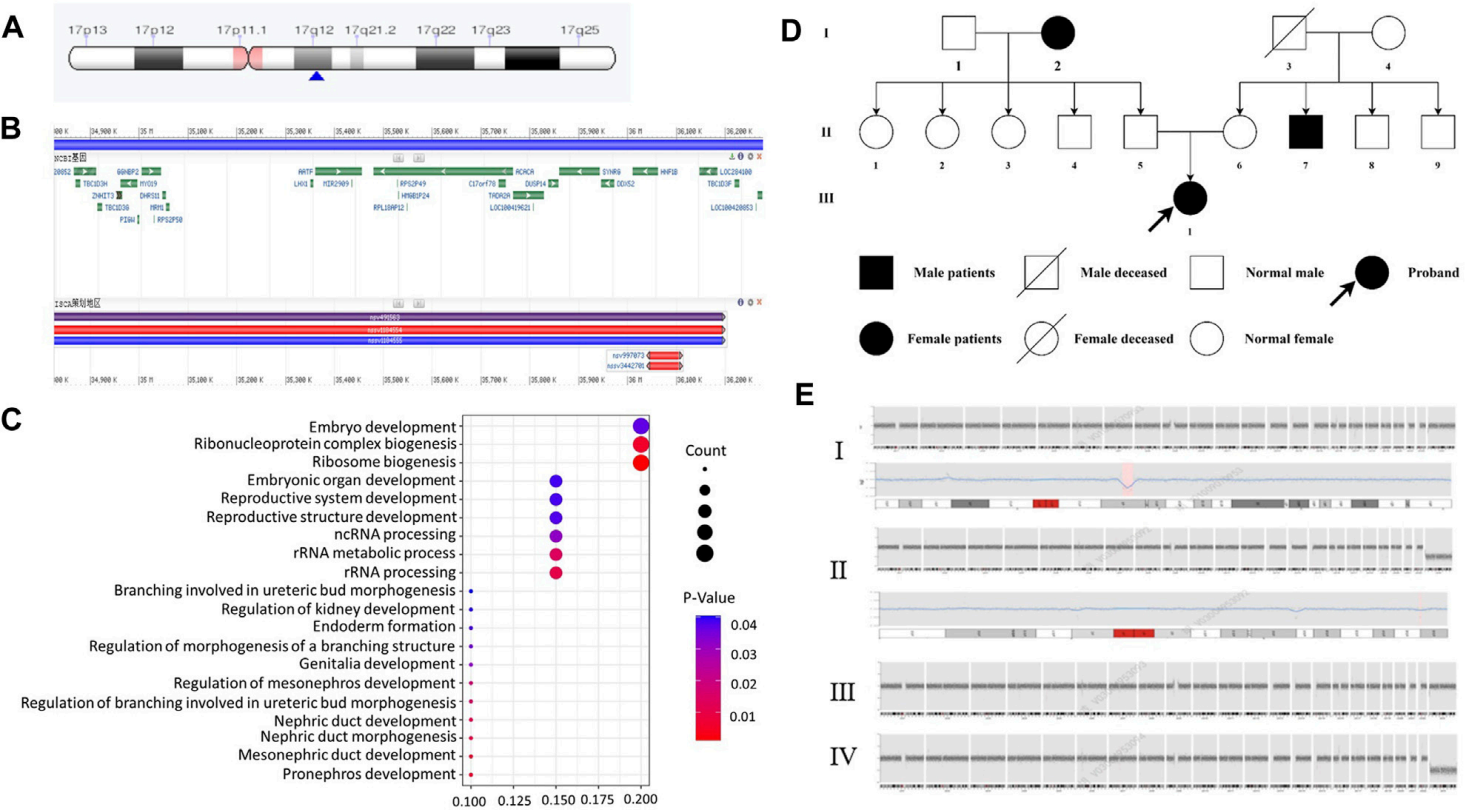
Genetic and chromosomal analyses. **(A)** Results of the CNV-Seq location of the missing fragment of the patient’s chromosome. The pink area is the mitotic site, and the blue arrow points the location of the patient’s missing gene fragment. **(B)** Results of CNV-Seq showed a 1.5-Mb deletion in the chr17: g.34825883_36276403 del region. **(C)** According to the results of enrichment analysis of Gene Ontology, we found, for the first time, that *LHX1* gene affects the urinary system and GGNBP2 and LHX1 genes affect the development of the female reproductive system. **(D)** Family tree of the proband. Squares: males; circles: females. White: no T2DM; black: T2DM and MODY-5. **(E)** CNV-seq results of family member. I: the patient with 1.5 Mb deletion in the chr17: g.34825883_36276403 del region. II: CNV-seq showed that the patient’s grandfather with 175 Kb deletion in the chr19: g.56217855_56392923del region. III-IV: no mutation in her mother and father.

We compared the C-peptide release and oral glucose tolerance test (OGTT) results conducted in 2020 and 2023 (Figures 1A, B). Based on these clinical presentations, we considered that whether there was a possibility of a special type of diabetes. Second-generation sequencing was performed using the genomic mNGS, copy number variation (CNV) sequencing technique. A 1.5-Mb deletion with haploinsufficiency for 20 genes within the 17q12 region was found (Figures 2A, B). The size of the deleted region and the number of deleted genes in the 17q12 region are similar with those reported cases for 1.40–1.94 Mb (Mitchel et al., 1993; Roehlen et al., 2018a; Jing et al., 2019; Cheng et al., 2022; Kumar et al., 2023). We performed Gene Ontology enrichment analysis on the deletion genes of the patients, and found that the deletion of different genes affected the development of different organs and systems, especially the LHX1 gene affected the development of the urinary system, and GGNBP2 and LHX1 jointly affected the development of the reproductive system (Figure 2C; Table 1). The patient’s other family members including her father, mother, and grandfather also underwent genetic testing; despite mutations, no mutations associated with this disease have been identified (Figure 2E). The patient currently was employed as a kindergarten teacher and exhibited reasonably cooperative and polite behavior. Her speech was spontaneous and normal in tone, rate, and volume. There were no apparent formal thought disorders. The patient had no problem with hearing, color vision, proteinuria, diabetic retinopathy, without intellectual disability, and any neuropsychiatric disorder.

**TABLE 1:** Biological roles of genes involved in GO enrichment analysis.

ID	Description	*p*-value	Genes
GO:0042254	Ribosome biogenesis	1.33E-03	*ZNHIT3, DDX52, AATF,* and *MRM1*
GO:0048793	Pronephros development	5.37E-03	*LHX1* and *HNF1B*
GO:0022613	Ribonucleoprotein complex biogenesis	5.63E-03	*ZNHIT3, DDX52, AATF,* and *MRM1*
GO:0072177	Mesonephric duct development	6.04E-03	*LHX1* and *HNF1B*
GO:0072178	Nephric duct morphogenesis	8.04E-03	*LHX1* and *HNF1B*
GO:0006364	rRNA processing	1.05E-02	*ZNHIT3, DDX52,* and *MRM1*
GO:0072176	Nephric duct development	1.07E-02	*LHX1* and *HNF1B*
GO:0016072	rRNA metabolic process	1.40E-02	*ZNHIT3, DDX52,* and *MRM1*
GO:0090189	Regulation of branching involved in ureteric bud morphogenesis	1.54E-02	*LHX1* and *HNF1B*
GO:0061217	Regulation of mesonephros development	1.74E-02	*LHX1* and *HNF1B*
GO:0034470	ncRNA processing	3.17E-02	*ZNHIT3, DDX52,* and *MRM1*
GO:0048806	Genitalia development	3.18E-02	*LHX1* and *HNF1B*
GO:0060688	Regulation of morphogenesis of a branching structure	3.51E-02	*LHX1* and *HNF1B*
GO:0009790	Embryo development	3.67E-02	*GGNBP2, LHX1, HNF1B,* and *AATF*
GO:0048608	Reproductive structure development	3.77E-02	*GGNBP2, LHX1,* and *HNF1B*
GO:0061458	Reproductive system development	3.83E-02	*GGNBP2, LHX1,* and *HNF1B*
GO:0001706	Endoderm formation	3.83E-02	*LHX1* and *HNF1B*
GO:0048568	Embryonic organ development	3.86E-02	*GGNBP2, LHX1,* and *HNF1B*
GO:0090183	Regulation of kidney development		*LHX1* and *HNF1B*
GO:0001658	Branching involved in ureteric bud morphogenesis	4.03E-02	*LHX1* and *HNF1B*

Through GO enrichment analyses, based on the National Library of Medicine database, HNF1B was confirmed to play important roles in the pathogenesis of 17q12 deletion syndrome. LHX1 deletion was found to contribute to urinary malformations and reproductive malformations. In addition, deletion of GGNBP2 contributed to reproductive malformations.

Regarding family history, the patient is an only child, and the grandparents (Figure 2D-I-2), uncle (Figure 2D-II-7), and grandfather (Figure 2D-I-3) were diagnosed with T2MD, and the grandfather has passed away (unknown cause of death). There was no history of consanguineous marriage in her family. Physical examination includes the following: 5.48 feet, body weight—50 kg, BMI—18 kg/m^2^, no purple striae, no acanthosis nigricans, and normal skin elasticity. For systemic examination, no problem was found in the heart, lungs, spine, limbs, and neuropsychiatric system. Her breasts (B4) and pubic hair development (Ph5) were normal (Tanner stage).

Biochemical parameter examination showed that cardiac biomarkers, B-type natriuretic peptide (BNP), tests for blood coagulation, fibrinolysis and autoimmune kidney disease, fecal routine test, thyroid function tests, urinary microalbumin/protein quantification tests, and gonadal hormone levels were within normal limits (Table 2).

**TABLE 2:** Biochemical parameter examination in the year 2023.

Biochemical parameter	Result	Normal range
HbA1c (%)	7.80	4–6
eGFR (mL/min/1.73m^2^)	124.45	56–122
Blood amylase (µg/L)	58.61	58.61
Urine amylase (U/L)	55.00	32–641
Magnesium (mmol/L)	0.47	0.75–1.02
FT4 (pmol/L)	18.4	12–22
TSH (mIU/L)	2.21	0.27–4.2
TPO (IU/mL)	<9.00	0–34
Anti-islet cell antibody	(−)	(−)
Anti-islet cell antibody IA-2A	(−)	(−)
Anti-insulin antibody	(−)	(−)
Anti-glutamic acid decarboxylase antibody	(−)	(−)

HbA1c, Glycosylated hemoglobin concentration A1c; eGFR, estimated glomerular filtration rate; FT4, free thyroxine; TSH, thyroid-stimulating hormone; TPO, thyroid peroxidase.

For genetic analysis, the venous blood of the patient and the family members was collected with the EDTA anticoagulation reagent. Using mNGS technology, CNV analysis was then performed. We detected seq [GRCh37] del (Cirio et al., 2024) (q12) chr17: g3825883-36276403 del, with a size of 1.5 Mb, containing a total of 20 genes, namely, *AATF*, *ACACA*, *C17orf78*, *DDX52*, *DHRS11*, *DUSP14*, *GGNBP2*, *HNF1B*, *LHX1*, *LHX1-DT*, *MIR2909*, *MIR378J*, *MRM1*, *MYO19*, *PIGM*, *SNORA90*, *SYNRG*, *TADA2A*, *YWHAEP7*, and *ZNHIT3* (Figures 2A, B, E-I). We then performed second-generation gene sequencing on the patient’s parents and grandfather, and the family members had no related gene mutations (Figure 2E-II, III, IV).

## 3 Discussion

The frequent misdiagnosis of MODY subtypes makes it necessary to perform genetic testing as early as possible so that accurate diagnosis and management plans can be introduced earlier. This case report highlights the typical clinical features of the patient diagnosed with 17q12 deletion syndrome including MODY5 and morphological abnormalities of the kidney. So all patients with suspected MODY5 should also be evaluated for common clinical features of 17q12 deletion syndrome (Roehlen et al., 2018b; Laffargue et al., 2024). Gene Ontology Overview of the deleted genes showed both HNF1B deletions and LHX1 deletions have significant impacts on structural or functional kidney dysfunctions and malformations of the reproductive system. In addition, GGNBP2 was also involved in reproductive system development.

Previous reports showed that HNF1B mutation contributed mainly to presentations of MODY5, including pancreatic hypoplasia, genital tract malformations, abnormal liver function, kidney dysfunction, learning disabilities, autism spectrum disorder, schizophrenia, müllerian abnormalities, and early-onset gout (Clissold RL et al., 2015; Bingham et al., 2024; Bockenhauer and Jaureguiberry, 2024; Clissold et al., 2024). This patient presented with multi-system abnormalities including DM, pancreas, kidney, and reproductive system abnormalities. It is different from typical MODY5; so are there other genes which act synergistically to cause these malformations?

Through bioinformatics analysis, LHX1 and GGNBP2 were maybe involved in the regulation of kidney development and the reproductive system. In the vertebrate embryo, the kidney is derived from the intermediate mesoderm. LHX1 is expressed early in the intermediate mesoderm and is one of the first genes to be expressed in the nephric mesenchyme. Experimental evidence suggests that embryos depleted of LHX1 show an almost complete loss of the kidney (Cirio et al., 2024). *LHX1* gene was considered a candidate gene responsible for renal hypoplasia and loss of the female genital tract in animal experiments (Kobayashi et al., 2024; Tam et al., 2024). Ledig et al. (2024) found that LHX1 heterozygous mutation causing congenital dysplasia of the uterus and upper vagina. Thus, in addition to HNF1B, LHX1 gene deletion was also contributed to dysfunction of the urinary and reproductive systems.

GGNBP2, also referred to ZFP403, encoded in the human chromosome 17q12-q23 has a single C2H2 zinc finger and a consensus LXXLL nuclear receptor-binding motif (Clissold et al., 2015). GGNBP2 played a key role in spermatogenesis by affecting the morphology and function of SOX9-positive Sertoli cells (Chen et al., 2017). There are few reports for the roles of this gene on the female reproductive system development. We speculate for the first time that the GGNBP2 gene may affect the development of the female reproductive system.

## 4 Summary

In addition to HNF1B absence, LHX1 and GGNBP2 absence was also involved in regulation of kidney development and the reproductive system through bioinformatics analysis in 17q12 deletion syndrome.

## Data Availability

The data that support the findings of this study are openly available in NCBI Sequence Read Archive at https://dataview.ncbi.nlm.nih.gov/object/PRJNA1146488?reviewer=oejtmrfk20gd5l7s1hnnrrs4on, reference number Bio Project: PRJNA1146488.
